# FABP4 reversed the regulation of leptin on mitochondrial fatty acid oxidation in mice adipocytes

**DOI:** 10.1038/srep13588

**Published:** 2015-08-27

**Authors:** Lu Gan, Zhenjiang Liu, Weina Cao, Zhenzhen Zhang, Chao Sun

**Affiliations:** 1 College of Animal Science and Technology, Northwest A&F University, Yangling, Shaanxi, 712100, China

## Abstract

Fatty acid binding protein 4 (FABP4), plays key role in fatty acid transportation and oxidation, and increases with leptin synergistically during adipose inflammation process. However, the regulation mechanism between FABP4 and leptin on mitochondrial fatty acid oxidation remains unclear. In this study, we found that FABP4 reduced the expression of leptin, CPT-1 and AOX1 in mice adipocytes. Conversely, FABP4 was down-regulated in a time-dependent manner by leptin treatment. Additionally, forced expression of FABP4 attenuated the expression of PGC1-α, UCP2, CPT-1, AOX1 and COX2 compared with leptin incubation. Moreover, mitochondrial membrane potential, fatty acid oxidation enzyme medium-chain acyl-CoA dehydrogenase (MCAD), long-chain acyl-CoA dehydrogenase (LCAD) and Cyt C levels were reduced in response to the overexpression of FABP4. These reductions correspond well with the reduced release of free fatty acid and the inactivation of mitochondrial complexes I and III by FABP4 overexpression. Furthermore, addition of the Akt/mTOR pathway-specific inhibitor (MK2206) blocked the mitochondrial fatty acid oxidation and respiration factors, whereas interference of FABP4 overcame these effects. Taken together, FABP4 could reverse the activation of the leptin-induced mitochondrial fatty acid oxidation, and the inhibition of Akt/mTOR signal pathway played a key role in this process.

Fatty acid binding protein 4 (FABP4) is a member of fatty acid binding protein family, and has been identified as peripheral membrane protein associated with a variety of hydrophobic compounds, such as long chain fatty acids, various cyclooxygenases and lipoxygenase metabolites^1^,^2^. FABP4 has been shown to be associated with lipid metabolism disorders, diabetes and obesity^3^,^4^. Other studies show the expression of FABP4 is increased during adipocytes differentiation, and the activity of FABP4 can be altered by long-chain fatty acids (LCFA), oxidized low-density lipoprotein (OX-LDL), peroxisomal proliferator-activated receptor γ (PPARγ) and insulin^5^. FABP4 is considered as a lipid companion for its characteristics of combining LCFA with high affinity to improve their solubility and shuttling them between the membranes and organelles^6^,^7^. Therefore, FABP4 affects the ingestion, transportation, esterification and β-oxidation of fatty acids, and regulates energy balance and lipid signal transduction in the body, such as carrying fatty acids to the lipid droplets for storage or to mitochondria and peroxisomes for oxidization. FABP4 also transports fatty acids to the endoplasmic reticulum and cell nucleus to regulate transcription and lipid-mediated signaling transference and to modulate the activity of enzyme and synthesis of biofilm^8^.

Leptin is a hormone produced by adipocytes, and plays a key role in the regulation of food intake and energy expenditure of animals. Chronic elevation of serum leptin stimulates fatty acid oxidation (FAO) and triglyceride (TG) hydrolysis, and reduces total FA uptake, resulting in induction of intramuscular TG in lean rats^9^,^10^,^11^. Mitochondria are a place where fatty acid oxidation, energy metabolism, intracellular oxidative phosphorylation and ATP synthesis take place, especially in adipose and muscle tissues. Dysfunction of mitochondria could induce obesity, cancer, neurodegenerative diseases and other metabolic disorders^12^,^13^,^14^,^15^. The number of mitochondria is increasing during the process of adipocyte differentiation, and FABP4 is a key factor in adipocyte differentiation^16^,^17^. However, the effect of FABP4 on mitochondrial development and function, especially in fatty acid oxidation, remains unclear in adipocytes. FABP4 purportedly plays physiological regulatory role opposite to leptin on fatty acid oxidation.

In this study, we demonstrated that FABP4 inhibited leptin-stimulated mitochondrial fatty acid oxidation, and phosphorylation inhibition of Akt/mTOR signal pathway was involved during this process. The results could provide a theoretical basis for the prevention and treatment of metabolic syndromes using FABP4.

## Results

### Inverse regulatory role of FABP4 and role of leptin on fatty acid oxidation

**Figure 1:**
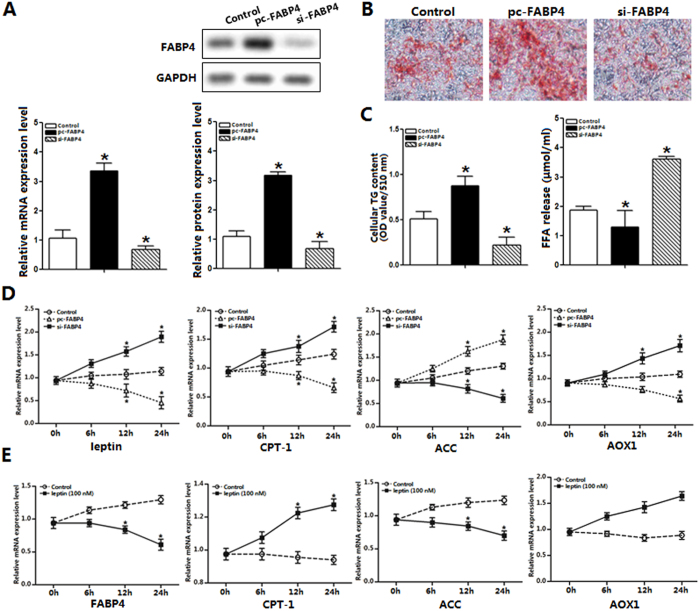
Inverse regulatory role of FABP4 and role of leptin on fatty acid oxidation. (**A**). The expression level of FABP4 after 48 h treatment with control vector, pc-FABP4, si-FABP4 (n = 6). (**B**). Images of adipocytes stained by Oil Red O staining with FABP4 transfection and differentiation until 6 day (n = 6). (**C**). Cellular TG content and FFA content after FABP4 transfection, differentiation on 6 day (n = 6). (**D**). The mRNA expressions of leptin, CPT-1, ACC and AOX1 after 0 h, 6 h, 12 h, 24 h of FABP4 transfection (n = 6). (**E**). The expression of FABP4, CPT-1, ACC and AOX1 after 0 h, 6 h, 12 h, 24 h of 100 nM leptin treatment (n = 6). pc-FABP4: FABP4 overexpression vector; si-FABP4: FABP4 shRNA vector; control: pcDNA 3.1-vector; CPT1: carnitine palmitoyl transferase-1, ACC: acetyl-CoA carboxylase, AOX1: acyl-coenzyme A oxidase; Scale bar, 100 μm; Values are means ± SD. vs. control group, *p < 0.05.

We first determined the transfection efficiency of FABP4. As expected, FABP4 was three times greater in FABP4 overexpression group compared with control group while decreased 55% after FABP4 was stable knocked down (Fig. 1A). Figure 1B,C showed forced expression of FABP4 significantly increased lipid accumulation (*p *< 0.05), whereas it reduced the expression of leptin in a time-dependent manner (*p *< 0.05) (Fig. 1D). Then we detected the expression of CPT1 and AOX1, as expected these two mitochondrial marker genes had a significantly decreased in Fig. 1D (p < 0.05). However the expression of ACC was elevated significantly (*p *< 0.05) (Fig. 1D). Along with these results, we observed that after cells were incubated with 100 nM leptin for 24 h, FABP4 protein expression was reduced (*p *< 0.05) in a time-dependent manner. Expression of CPT-1 (*p *< 0.05) and AOX1 (*p *< 0.05) were induced while ACC was down-regulated (Fig. 1E). From these results we established that FABP4 and leptin play opposite roles in the regulation of fatty acid oxidation.

### FABP4 reversed the promoting functions of leptin on fatty acid oxidation

**Figure 2:**
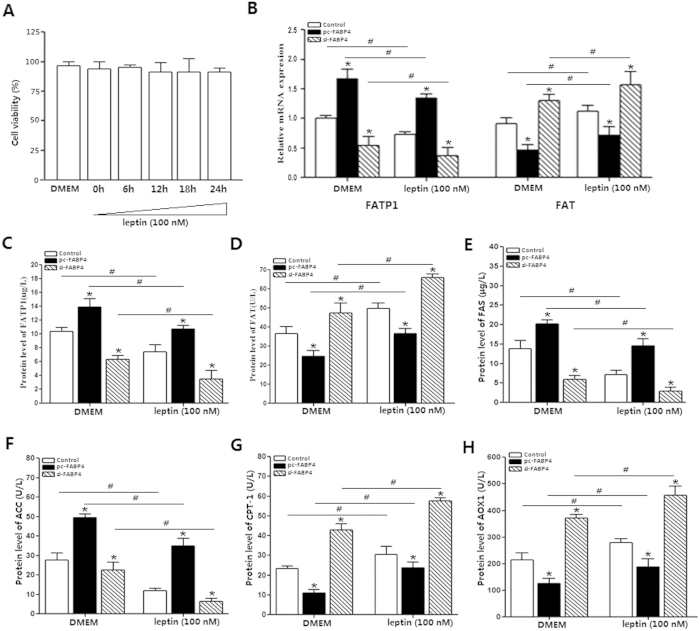
FABP4 reversed the promoting functions of leptin on fatty acid oxidation. The DMEM was used as the control against leptin treatment and cells were incubated with 100 nM leptin for 24 h or without leptin treatment. (**A**). Cell viability was detected by CCK-8 kit after 100 nM leptin treatment for 0 h, 6 h, 12 h, 18 h and 24 h (n = 6). (**B**). The mRNA expression of adipogenesis genes FATP1 and FAT after transfection with control vector, pc-FABP4 and si-FABP4 (n = 6). (**C**). Protein expression of FATP after transfected with control vector, pc-FABP4 and si-FABP4 (n = 6). (**D**). Protein expression of FAT after transfection with control vector, pc-FABP4 and si-FABP4 (n = 6). (**E**). Protein expression of FAS after transfected with control vector, pc-FABP4 and si-FABP4 (n = 6). (**F**). Protein expression of ACC after transfected with control vector, pc-FABP4 and si-FABP4 (n = 6). (**G**). Protein expression of CPT-1 after transfected with control vector, pc-FABP4 and si-FABP4 (n = 6). (**H**). Protein expression of AOX1 after transfection with control vector, pc-FABP4 and si-FABP4 (n = 6). All the protein levels were detected by ELISA test; pc-FABP4: FABP4 overexpression vector; si-FABP4: FABP4 shRNA vector; control: pcDNA 3.1-vector; FATP1: Fatty acid transport protein1, FAS: fatty acid synthase, CPT-1: carnitine palmitoyl transferase-1, ACC: acetyl-CoA carboxylase, AOX1: acyl-coenzyme A oxidase; Values are means ± SD. vs. control group, *p < 0.05, ^#^*p < *0.05.

In this study, ELISA test was used to detect the key enzymes of fatty acid synthesis (FATP, ACC and FAS), and the key enzymes of fatty acid mobilization (FAT, AOX1), and fatty acid oxidation rate-limiting enzyme, CPT-1. In Fig. 2A, we showed 100 nM leptin treatment did not alter the cell viability after 24 h treatment (*p *> 0.05). To further examine the interaction between FATP1, FAT and FABP4 on promoting free fatty acids absorption of adipocytes, we measured the expression of FATP1 and FAT by real-time PCR. The results (Fig. 2B) showed the mRNA expression level of FATP1 was increased (*p *< 0.05) and the FAT expression level was decreased in respond to FABP4 overexpression. After leptin treatment the expression level of FATP1 was attenuated and FAT expression was increased in contrast. Figure 2C–H showed the positive regulation role of leptin on lipid oxidation. The increased levels of FATP, ACC and FAS by forced expression of FABP4 were attenuated after leptin treatment (*p *< 0.05). Moreover, FABP4 also reduced the levels of FAT, CPT-1 and AOX1, and decreased fatty acid mobilization and oxygenolysis (*p *< 0.05). Leptin treatment attenuated these effects. Thus, our results suggested that FABP4 reversed the positive effect of leptin on adipocyte fatty acid oxidation and effectively blocked leptin function in these assays.

### FABP4 accentuated oligomycin-induced mitochondrial dysfunction

**Figure 3:**
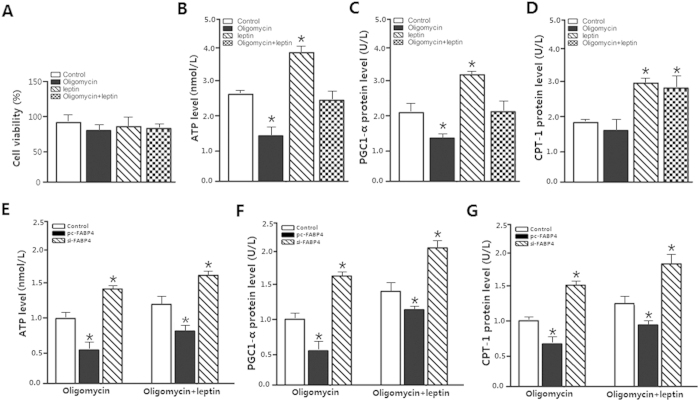
FABP4 accentuated oligomycin-induced mitochondrial dysfunction. (**A**). Cell viability was detected by CCK-8 kit after 100 nM leptin treatment for 24 h and 1 μM oligomycin treatment 1 h (n = 6). (**B**). ATP level with 100 nM leptin treatment for 24 h and 1 μM oligomycin treatment 1 h (n = 6). (**C**). PGC1-α protein level after 100 nM leptin treatment for 24 h and 1 μM oligomycin treatment 1 h (n = 6). (**D**). CPT-1 protein level with 100 nM leptin treatment for 24 h and 1 μM oligomycin treatment 1 h (n = 6). (**E**). ATP level after transfection with FABP4, pre-incubated cells with 1 μM oligmycin for 1 h or 100 nM leptin for 24 h (n = 6). (**F**). PGC1-α protein level after transfection with FABP4, pre-incubated cells with 100 nM leptin for 24 h or 1 μM oligomycin for 1 h (n = 6). (**G**). CPT-1 protein level after transfection with FABP4, pre-incubated cells with 1 μM oligmycin for 1 h or 100 nM leptin for 24 h (n = 6). All the protein levels were detected by ELISA test; pc-FABP4: FABP4 overexpression vector; si-FABP4: FABP4 shRNA vector; control in (**D–F**): pcDNA 3.1-vector; CPT-1: carnitine palmitoyl transferase-1.Values are means ± SD. vs. control group, *p < 0.05.

Oligomycin is an inhibitor of respiratory-chain phosphorylation; it caused dysfunction of mitochondrion respiration^18^. To determine how FABP4 affected mitochondrial oxidation phosphorylation, we used leptin and oligomycin incubated cells. Cell viability did not change (*p *> 0.05) (Fig. 3A). Leptin alone remarkably increased the levels of ATP (*p *< 0.05), PGC1-α (*p *< 0.05) and CPT-1 (*p *< 0.05) (Fig. 3A–C), improving the mitochondrial oxidation function. Oligomycin dramatically decreased the levels of mitochondrial ATP and PGC1-α (*p *< 0.05), while CPT-1 expression level was not significantly different (*p *> 0.05). The forced expression of FABP4 exacerbated the impairment of mitochondrial respiration in both oligomyclin treatment (*p *< 0.05) and oligomyclin plus leptin treated groups (*p *< 0.05), whereas stable knocked down FABP4 elevated the ATP, PGC1-α and CPT-1 levels (*p *< 0.05) (Fig. 3D–F). These results indicated FABP4 had a negative effect while leptin had a positive effect on mitochondrial respiration function. And, leptin could be used as an enhancer for mitochondrial function.

### FABP4 decreased mitochondrial fatty acid oxidation-related genes expression

**Figure 4:**
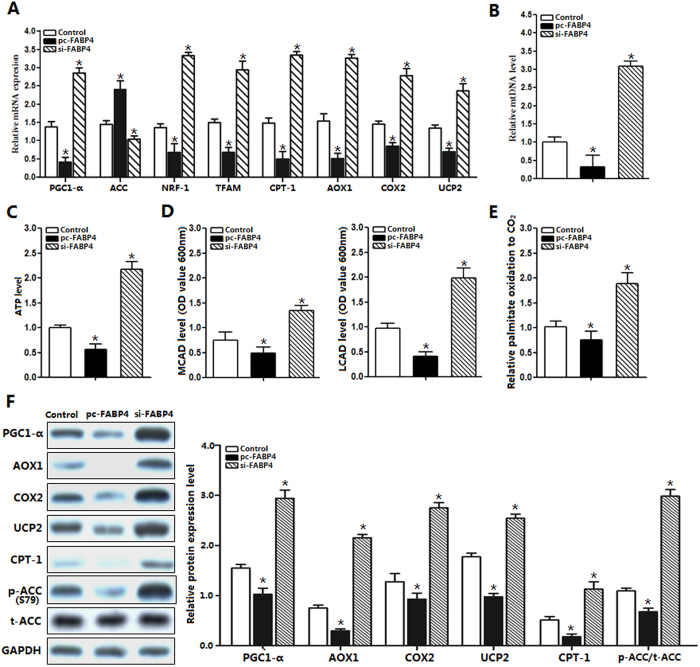
FABP4 decreased mitochondrial fatty acid oxidation-related genes expression. (**A**). mRNA expressions of the mitochondrial key genes after transfection with FABP4, pre-incubated cells with 100 nM leptin for 24 h (n = 6). (**B**). mtDNA copy number after transfection with FABP4 plasmids, pre-incubated cells with 100 nM leptin for 24 h (n = 6). (**C**). ATP level with FABP4 treatment, pre-incubated cells with 100 nM leptin for 24 h (n = 6). (**D**). MCAD and LCAD protein levels after transfection with FABP4, pre-incubated cells with 100 nM leptin for 24 h (n = 6). (**E**). Fatty acid oxidation. Cells were infected with FABP4 plasmids, and palmitate oxidation to CO_2_ was measured for 3 h (n = 6). (**F**). Protein expression of PGC-1α, AOX1, COX2, UCP2, CPT-1, ACC and p-ACC^Ser79^ after transfection with FABP4, pre-incubated cells with 100 nM leptin for 24 h (n = 6). The level of total GAPDH was determined as loading control. pc-FABP4: FABP4 overexpression vector; si-FABP4: FABP4 shRNA vector; control: pcDNA 3.1-vector. Values are means ± SD. vs. control group, *p < 0.05.

To further explore the impact of FABP4 on mitochondrial oxidative and respiratory functions in adipocytes, cells were incubated with 100 nM leptin for 24 h and measured the marker genes of mitochondrial oxidation. Compared with those in the control group, expression of *PGC1-α, NRF-1, TFAM, CPT-1, AOX1, COX2* and *UCP2* were all significantly down-regulated in FABP4 forced expression group (*p *< 0.05), while *ACC* was elevated (Fig. 4A). At the same time, mitochondial DNA copy number was reduced (*p *< 0.05) along with the lowered ATP level after FABP4 forced expression (*p *< 0.05; Fig. 4B–C). Additionally, the key enezymes of mitochondrial fatty acid oxidation medium chain acyl-CoA dehydrogenase (MCAD) and long-chain acyl-CoA dehydrogenase (LCAD) expressions were both decreased in FABP4 overexpression group (*p *< 0.05, Fig. 4D). FABP4 overexpression cells produced a 1.8-fold increase in palmitate oxidation to CO_2_ compared with control group (Fig. 4E). Figure 4F indaiceted forced expression of FABP4 reduced the protein levels of AOX1, COX2, UCP2, CPT-1 and p-ACC. These data demonstrated that FABP4 is a negative regulator of mitochondrial fatty acid oxidation.

### FABP4 reduced mitochondrial respiratory activity memberane potential

**Figure 5:**
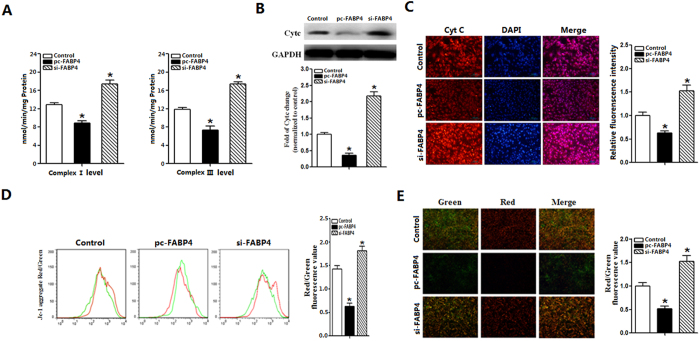
FABP4 reduced mitochondrial respiratory activity membrane potential. (**A**). The activities of mitochondrial complex I and III after transfection with FABP4, pre-incubated cells with 100 nM leptin for 24 h (n = 6). (**B**). The expression of Cyt C after transfection with FABP4, pre-incubated cells with 100 nM leptin for 24 h (n = 6). (**C**). Images of adipocytes Cyt C immunofluorescent staining after transfection with FABP4, pre-incubated cells with 100 nM leptin for 24 h, Red Scale bar: 100 μm (n = 6). (**D**). Cells were analyzed by flow cytometry with JC-1stainning after transfection with FABP4, pre-incubated cells with 100 nM leptin for 24 h (n = 6). (**E**). Images of adipocytes stained with immunofluorescent of JC-1 under a fluorescence microscope after transfected with FABP4, pre-incubated cells with 100 nM leptin for 24 h, Scale bar: 100 μm (n = 6). The level of total GAPDH was determined as loading control. pc-FABP4: FABP4 overexpression vector; si-FABP4: FABP4 shRNA vector; control: pcDNA 3.1-vector. Values are means ± SD. vs. control group, *p < 0.05.

We explored whether FABP4 affected other mitochondrial functions. Adipocytes were pretreated with leptin (100 nM) and were harvested 5 days after transfection with FABP4. We measured the respiratory activity first. Activities of mitochondrial complexes I and III were both reduced in FABP4 overexpression group, but enhanced substantially by silenceing FABP4 (*p *< 0.05; Fig. 5A). Cyt C content and mitochondrial membrane potential were studied by immunofluorescent staining and JC-1 staining. Result showed fluorescence intensity of Cyt C in FABP4 group was lower than that of control group (Fig. 5C). Real-time and western blot analysis of Cyt C verified that FABP4 decreased the expression of Cyt C (*p *< 0.05; Fig. 5B). As shown in Fig. 5E, FABP4 alleviated red fluorescence intensity, and diminshed the ratio of the red/green light by 50%, while silenceing FABP4 gene elevated the red/green ratio (*p *< 0.05; Fig. 5D).

### Akt/mTOR pathway was involved in FABP4 regulation of mitochondrial fatty acid oxidation

**Figure 6:**
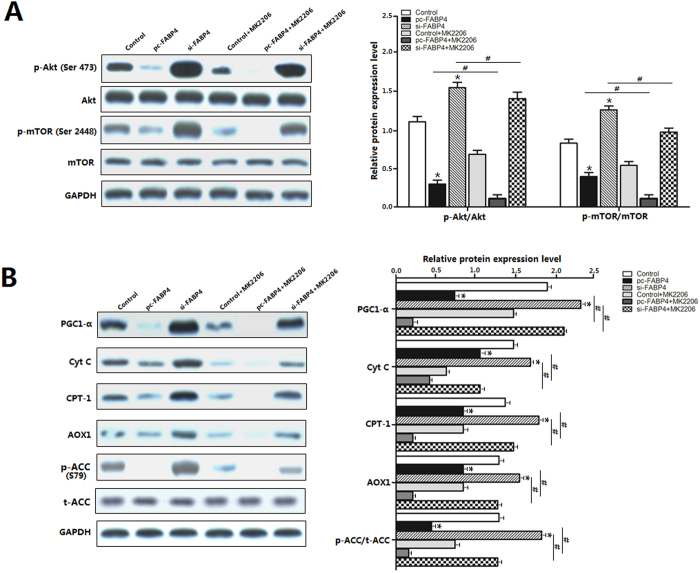
Akt/mTOR pathway involved in FABP4 regulation of mitochondrial fatty acid oxidation. Adipocytes were pretreated with 100 nM leptin or MK2206, and then transfected with FABP4 plasmids. (**A**). Representative immunoblots and densitometric quantification for p-Akt^ser473^, total Akt, p-mTOR^Ser2448^ and total mTOR (n = 6). (**B**). Representative immunoblots and densitometric quantification for PGC1-α, AOX1, Cyt C, CPT-1, ACC and p-ACC^Ser79^ (n = 6). The level of total GAPDH was determined as loading control. pc-FABP4: FABP4 overexpression vector; si-FABP4: FABP4 shRNA vector; control: pcDNA 3.1-vector. Values are means ± SD. vs. control group, *p < 0.05, ^#^*p < *0.05.

To further characterize the underlying mechanisms for the regulation of FABP4 on mitochondrial fatty acid oxidation, we evaluated the Akt/mTOR signal pathway after leptin incubation. The ratio of phosphorylated Akt^ser473^ to the total Akt and phosphorylated mTOR^Ser2448^ to the total mTOR were both elevated (*p *< 0.05) by knock down FABP4 (Fig. 6A). Interestingly, we noticed the levels of PGC1-α, Cyt C and CPT-1 were also elevated (*p *< 0.05) in Fig. 6B. Conversely, suppression of Akt pathway by the Akt specific inhibitor MK2206 decreased (*p *< 0.05) Akt phosphorylation and also reduced mTOR (*p *< 0.05) phosphorylation (Fig. 6A). However, interference of FABP4 attenuated the effects of MK-2206, and alleviated the expression of PGC1-α, Cyt C, AOX1 and CPT-1 (*p *< 0.05). Finally, forced expression of FABP4 stimulated cell lipid accumulation (Fig. 6B) along with the reduced phosphorylation level of ACC1^Ser79^ (*p *< 0.05). These results implied that FABP4 regulated mitochondrial fatty acid oxidation via Akt/mTOR signal pathway.

## Discussion

FABP family plays an important role in regulating fatty acids trans-membrane transportation, and these proteins establish the fatty acid transport system, mediating and promoting absorption of free fatty acids^10^. FABP4 promotes fatty acid accumulation at cell surface and forms a concentration gradient within cell membranes, and combines long-chain unsaturated fatty acids specifically by its high affinity capacity to deliver them into mitochondria and other organelles^19^,^20^. Leptin enhances FA flux into the mitochondria by increasing the expression of CPT-1 or by promoting its activity, possibly through a reduction in ACC and finally reducing malonyl-CoA^21^,^22^. In the present study we illustrated a negative interaction between FABP4 and leptin as well as their opposing regulatory role on mitochondrial fatty acid oxidation key factors CPT-1, ACC and AOX1. Moreover, FABP4 increased the expression of FATP1 and reduced FAT expression, indicating that FABP4 enhanced the fatty acids transportation. Conversely, leptin alleviated this effect. We also found FABP4 activated fatty acid synthesis key enzymes FAS and inhibited phosphorylation of ACC, meanwhile impaired the activity of fatty acid oxidation key enzymes CPT-1 and AOX1.

PGC1-α is a key regulator of mitochondrial energy metabolism and biosynthesis from enhancing mitochondrial respiratory, fatty acid β-oxidation, the Krebs cycle and oxidative phosphorylation to stimulating the enzyme activity and promoting the mitochondrial biogenesis^23^. Decreasing PGC1-α expression will reduce insulin signaling molecules activity, leading to insulin resistance^24^. We showed in this study that both oligomycin and leptin reduced the activity of PGC1-α as accompanied with reduction of ATP production in adipocytes. These results further proved that leptin is an agonist of mitochondrial fatty acid oxidation. Mitochondrial number and function are altered in response to external stimuli in eukaryotes^25^. The role of mitochondria in lipid homeostasis has been strongly emphasized in some recent studies focusing on mitochondrial respiratory deficiency^26^,^27^. Our results indicated that FABP4 inhibited the expressions of mitochondrial metabolic factors including PGC1-α, NRF-1, TFAM, and UCP2. FABP4 also attenuated the activity of fatty acid oxidation key enzymes MCAD, LCAD, CPT-1, AOX1 and COX2, whereas the reversal effects of the silencing FABP4 reinforced the roles of FABP in lipid metabolism. Additionally, mitochondrial content was decreased by FABP4 along with the declined of ATP level.

Mitochondrial electron transport chain (mETC) is composed of four complexes (complexes I, II, III and IV). mETC is built to accept electrons from NADH and FADH2, transfer them through a series of redox reactions to molecular oxygen to produce H_2_O, and simultaneously couple this exergonic reaction to the translocation of protons across the inner membrane^12^. At the same time, due to the generation of free radicals, the respiratory chain and oxidative phosphorylation are uncoupled, calcium homeostasis is disordered and cells are damaged, triggering apoptosis^28^,^29^. We found FABP4 attenuated the activities of complexes I and III, inhibited the expression of Cyt C. Moreover, the membrane potential was reduced by FABP4 treatment. Mitochondrial membrane potential down-regulating shows that mitochondrial permeability transition pore (PT) was opened, the mitochondrial membrane permeability is increased, and cellular energy metabolism is impaired^30^,^31^. Thus we inferred that FABP4 inhibited the mitochondrial electron transport chain and energy metabolism. This led to the alternation of fatty acid oxidation.

A possible role of the Akt/mTOR signaling network is associated with fat metabolism has been proposed recently. In particular, mTOR appears to play an important role in adipogenesis as rapamycin treatment prevents adipocyte differentiation and lipid accumulation^32^. The regulation of fat metabolism via Akt/mTOR is also related to a decrease in fat accumulation due to enhanced β oxidation^33^. Our results in this study suggested that FABP4 inhibited the phosphorylation of Akt/mTOR signal pathway, and it also suppressed mitochondrial fatty acid oxidation as evidenced by the reductions of PGC1-α, CPT-1, Cyt C and AOX1. However, FABP4 knock down with MK2206 treatment, a specific inhibitor of Akt/mTOR signal pathway, attenuated those effects significantly. The results confirmed that Akt/mTOR signaling pathway was necessary for FABP4 to block mitochondrial fatty acid oxidation.

**Figure 7:**
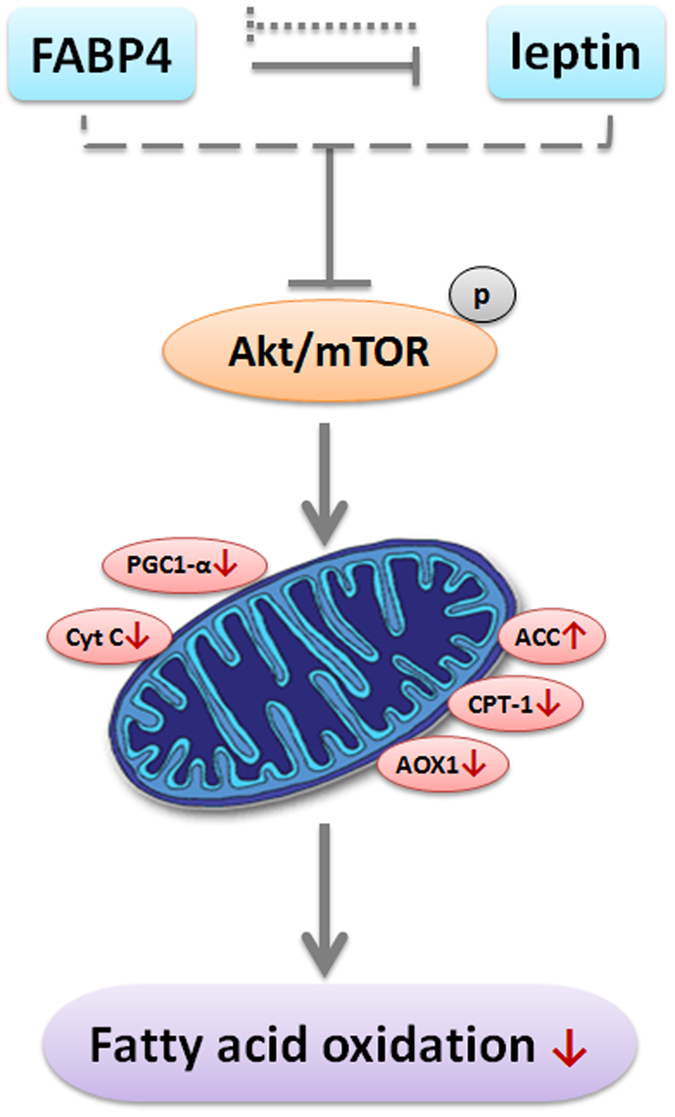
Summary of FABP4 and leptin in the regulation of mitochondrial fatty acid oxidation via Akt/mTOR signaling pathway in mice adipocytes. FABP4 and leptin play opposite role in mitochondrial fatty acid oxidation. *Our co-author Zhenjiang Liu designed and drew this Figure.*

In summary, our study provided a new insight into the mechanisms required for the regulation of FABP4 on adipocyte mitochondrial fatty acid oxidation (Fig. 7). Additionally, we identified that FABP4 reversed the up-regulate role of leptin on mitochondrial fatty acid oxidation by inhibiting Akt/mTOR signal pathway. These results will be valuable in developing of prevention and treatment for obesity and type II diabetes.

## Materials and Methods

### Mice adipocyte culture

Two-week-old male Kunming mice were purchased from the Laboratory Animal Center of the Fourth Military Medical University, China. All mice experiments were carried out in accordance with the protocol approved by the Animal Ethics Committee of Northwest A&F University and the experimental protocol was performed in accordance with applicable guidelines and regulations. Mice were allowed *ad libitum* access to water and standard laboratory diet and kept in the animal room that was maintained at constant temperature at 25 °C ± 1 °C, humidity at 55 ± 5%, and a 12 h light/12-dark cycle.

Epididymal white adipose tissues were dissected out, visible fibers and blood vessels were removed, and adipose tissue was washed three times with PBS buffer containing 200 U/mL penicillin and 200 U/mL streptomycin (Sigma, St. Louis, USA). Then the adipose tissue was minced into fine sections (1 mm^3^) with scissors and incubated in 10 mL of digestion buffer containing Dulbecco’s modified eagle medium (DMEM)/F-12 (Gibco, USA), 100 mM HEPES (Sigma, St. Louis, USA), 1.5% bovine serum albumin (Sigma, St. Louis, USA), 2 mg/mL type I collagenase (Sigma, St. Louis, USA) for 50 min at 37 °C in a water bath. After the incubation, growth medium (DMEM/F-12 (50:50)), 10% fetal bovine serum (Sigma, St. Louis, USA), 100 U/mL penicillin and 100 U/mL streptomycin were added to the digestion flask. Flask contents were mixed and filtered through nylon screens with 250 μm and 20 μm mesh openings to remove undigested tissue and large cell aggregates. Filtered cells were centrifuged at 1,300 × *g* for 7 min at room temperature to separate floating adipocytes from stromal-vascular cell pellets. Isolated cell pellets were suspended in DMEM/F12 (Invitrogen, USA). The cells were then seeded on 35-mm primary culture dishes at a density of 8 × 10^4^ cells/dish and incubated at 37 °C under a humidified atmosphere of 5% CO_2_ and 95% air until confluence.

### Transfection of adipocytes with si-FABP4 or pc-FABP4

The si-FABP4 plasmid was designed and synthesized commercially (GenePharma, China). To generate the pcDNA3.1-FABP4 (pc-FABP4), full-length mice FABP4 was released via KpnI and NheI digestion of the pMD18T-FABP4 and sub-cloned into pcDNA3.1 (Invitrogen, USA). And pcDNA-3.1-vector was used as the control. The X-treme GENE HP Reagent (Roche, Switzerland) was used in plasmids transfection. 2 μg DNA was mixed with Opti-MEM^®^ media (Invitrogen, USA) and X-treme GENE HP Reagent. The transfection mixture was added to each dish mentioned above afterwards. 48 hours after transfection the control vector efficiency, interference efficiency and over-expression efficiency were detected by real-time PCR.

### Treatments with leptin and oligomycin

After transfection with FABP4 reconstructed vectors for 2 days, cells were washed with phosphate-buffered saline (PBS) twice. Then cells were then incubated in serum-free medium for 90 min before 100 nM leptin (Sigma, USA) was added. After 6 h, 12 h, 18 h, and 24 h cells were washed once with ice-cold PBS and then cells were harvested separately for further study. 1 μM Oligomycin (Sigma, USA) was performed to incubate cells for 1 h according to the same protocol as leptin.

### Cell viability assay

Cell viability was determined by using by Cell Counting Kit 8 (CCK-8, Vazyme, China) assay according to the instructions. The transfected cells were seeded in 96-well plate at a density of 2 × 10^5^ and cultured for 12 h. 10 μl CCK-8 solution was added into each well and incubated for 1 hour at 37 °C. Absorbance was quantified at 450 nm by Vector 5 (Bio-Tech Instruments, USA).

### Cell Lipid measurement

Cells were washed three times in PBS buffer and then fixed in 10% (v/v) formaldehyde for 30 min. The fixed cells were then washed with PBS three times and stained with a working solution of Oil Red O for 30 min at room temperature. Cells were washed with deionized water and the images of the cells were observed with a Nikon TE2000-U Fluorescence Microscope (Tokyo, Japan). Then Oil Red O was extracted into 100% avantin for colorimetric analysis of triglyceride (TG) at 510 nm. Free cell culture medium was removed at day 8, and used to determine free fatty acid (FFA) content using FA Assay kit (Jiangcheng, China) at wave length 570 nm. The triglyceride concentration in this medium was determined using Infinity Triglyceride kit (Sigma, USA).

### Cell fatty acid oxidation measurement

Palmitate oxidation to CO_2_ and the incorporation of palmitate into lipids were measured according previous method^34^. Adipocytes were washed in Krebs-Ringer bicarbonate HEPES buffer (KRBH buffer: 135 mM NaCl, 3.6 mM KCl, 0.5 mM NaH_2_PO_4_, 0.5 mM MgSO_4_, 1.5 mM CaCl_2_, 2 mM NaHCO_3_, and 10 mM HEPES, pH 7.4) that contained 0.1% BSA, pre-incubated at 37 °C for 30 min in KRBH 1% BSA and washed again in KRBH 0.1% BSA. Cells were then incubated for 3 h at 37 °C with fresh KRBH containing 1 μCi/ml [1-^14^C] palmitate (Perkin Elmer, USA) bound to 1% BSA. Oxidation measurements were performed by trapping the radioactive CO_2_ in a parafilm-sealed system. The reaction was stopped by the addition of 40% perchloric acid through a syringe that pierced the parafilm.

### ELISA for fatty acid transporters

The cells pretreated with leptin and normal media were collected after si-FABP4 and pc-FABP4 transfection 96 h and then cells were disrupted by ultrasonication (28 KHz, 30 min). The contents of fatty acid transporters in the cell lysate were determined using commercial ELISA kits (R&D Systems, USA).

### Mitochondrial respiratory activity

Adipocyte mitochondria were isolated using the Cell Mitochondria Isolation kit (Beyotime, China). Cells were harvested and washed with cool-PBS twice, and then suspended in the ice-cold isolation buffer for 15 min. After the cells were homogenized, the homogenate was centrifuged at 1,000 × g for 10 min at 4 °C. The supernatant was collected and centrifuged at 11,000 × g for 10 min at 4 °C. The mitochondria were collected in the sediments. The activities of the mitochondrial complexes were determined using the Mito Complex I and III Activity Assay kits (GenMed Scientifics Inc., China).

### Mitochondrial content and mitochondrial damage assay

Fluorescent probe JC-1 (Beyotime, China) was used to estimate mitochondrial membrane potential. Briefly, cells were incubated with 5 μg/mL JC-1 at 37 °C for 10 min, then washed twice with PBS and placed in fresh medium without serum. Images of the cells were scanned by a Fluorescence Microscope (Nikon TE2000-U, Japan). At the same time, cells were gently harvested with trypsin, and transferred on ice to the flow cytometer. JC-1 was excited at 488 nm and the monomer signal (green) was recorded at 525 nm (JC-1 monomer) on a flow cytometer using a minimum of 10,000 cells per sample. Simultaneously, the aggregate signal (red) was recorded at 590 nm (JC-1 aggregates). The ratio of red/green fluorescent intensity was calculated.

Immunofluorescence analysis of CytC was performed 48 h after plasmids transfection, cells were washed three times with PBS, and fixed with 10% neutral formalin for 30 min and washed with PBS, then incubated with the rabbit against rat Cyt C antibody (Boster Biological Technology Co., China) (diluted 1:100 in PBS) for 12 h at 4 °C. After the incubation, cells were washed twice with PBS for 3 min, and then incubated with fluorescein isothiocyanate-conjugated goat against rabbit IgG antibody (Boster, China) (diluted 1:100 in PBS) for 1 h at room temperature, and washed again in PBS. Finally the cells were illuminated with the appropriate laser line and photographed with a TE2000 Nikon fluorescence microscopy (excitation filter BP 450–490, a beam splitter FT510 and an emission filter LP520, Tokyo, Japan).

Relative amounts of mtDNA copy number and nuclear DNA copy number were detected using QPCR method. Pairs of primers for the COX2 mtDNA and nDNA 18S rRNA was from our laboratory. The QPCR system was performed according to the instructions. After treatment with the indicated plasmids, ATP concentration was determined using the Luciferase-based ATP-assay kit from Roche (Roche, Switzerland).

### Real-time PCR analysis

Total RNA was extracted from cells by using RNAiso Plus Reagent (TaKaRa, Dalian, China) and used for synthesized of the first strand cDNA with PrimeScript^®^ RT reagent Kit (TaKaRa, Dalian, China). Primers for *FABP4, CPT-1(carnitine palmitoyl transferase-1), TFAM (mitochondrial transcription factor A), PGC1-α (PPARγ coactivator 1-α), NRF-1(nuclear respiratory factor-1), ACC (acetyl-CoA carboxylase), Cyt C (cytochrome C), COX2 (cyclo-oxyge-nase2), FATP1 (fatty acid transport protein1), FAT (fatty acid translocase), UCP2 (uncoupling protein 2), AOX1 (acyl-coenzyme A oxidase)*, and *β-actin* were designed by Premier 5.0 software. Primer sequences and amplification conditions were given in Table 1. β-a*ctin* was used as the internal control. Real-time PCR was performed with an iQ5 system (BioRad, USA) using a 20 μL reaction mixture containing 12.5 μL SYBR *Premix Ex Taq*™ II (TaKaRa, Japan), 1 μL Forward primer, 1 μL Reverse primer, 2 μL template cDNA, and 8.5 μL ddH_2_O. Real-time PCR amplification reactions were carried out on a Bio-Rad iQ5 by SYBR Premix Ex TaqTMII chemistry detection under amplification conditions. 2^−△△Ct^ method was chose to analysis the data (△Ct = Ct was for target gene, Ct for reference gene, △△Ct = △Ct was for treat group –△Ct for control group).

### Immunoblot analysis

Cells were lysed in the lysis buffer containing 20 mM Tris (pH 7.5), 5 mM EGTA, 150 mM NaCl, 1% Nonidet P-40, 0.1 mM Na_3_VO_4_, 1 mM NaF, 10 mM sodium β-glycerophosphate, 0.1 mM phenylmethylsulfonyl fluoride, 1 μg/mL leupeptin, 10 μg/mL aprotinin for 40 min at 4 °C. Removing insoluble material by centrifugation at 12,000 × g for 15 min at 4 °C, and the supernatants were used to assay protein levels. Protein samples (25 μg) were separated by electrophoresis on 12% and 5% SDS-PAGE gels using slab gel apparatus and then transferred to PVDF nitrocellulose membranes (Millipore, USA) blocked with 5% Skim Milk Powder/Tween 20/TBST at room temperature for 2 h. Primary antibodies against FABP4 and GAPDH were purchased from Bioword (USA) and against COX2, PGC1-α, CPT-1, ACC, phospho-ACC (Ser79), AOX1, UCP2 and Cyt C were form Abcam (USA) and against Akt, phospho-Akt (Ser473), mTOR, phospho-mTOR (Ser2448) were from Cell Signaling (USA). Akt specific inhibitor MK-2206 was from Abcam (USA). Membranes were incubated with the primary antibodies at 4 °C overnight and further incubated with the appropriate HRP-conjugated secondary antibodies (Boaoshen, China) for 2 h at room temperature. Proteins were visualized using chemiluminescent peroxidase substrate (Millipore, USA), and then the blots were quantified using ChemiDoc XRS system (Bio-Rad, USA) and Quantitative analysis of the immune-blotted bands was performed using Quality One software (Bio-Rad, USA).

### Statistical analysis

Statistical analyses were conducted using SAS v8.0 (SAS Institute, Cary, NC). Data were analyzed using one-way or two-way ANOVA. Comparisons among individual means were made by Fisher’s least significant difference (LSD). Data were presented as mean ± SD. *p *< 0.05 was considered to be statistically significant.

## Additional Information

**How to cite this article**: Gan, L. *et al.* FABP4 reversed the regulation of leptin on mitochondrial fatty acid oxidation in mice adipocytes. *Sci. Rep.* **5**, 13588; doi: 10.1038/srep13588 (2015).

## Figures and Tables

**Table 1:** Primers used for PCR.

*Gene*	*RT-PCR primers (5′-3′)*	*Tm (°C)*	*Accession No.*
*CPT-1*	F^a^: CTTCCAAGGCAGAAGAGTGGG	60.2	NM_013495.2
	R^b^: GAACCTTGGCTGCGGTAAGAC		
*PGC1-α*	F: CATGGATGGCCTATTTGATGAC	58.3	NM_133249
	R: CACGGAGAGTTAAAGGAAGAGC		
*NRF-1*	F: GCACCTTTGGAGAATGTGGT	56.5	BC005410
	R: GGGTCATTTTGTCCACAGAGA		
*AOX1*	F: CTGGAGCCCGCTTTGTCTT	56.6	NM_001271898.1
	R: CCTCGCCTTTCTTGATGGA		
*Cyt C*	F: AAATCTCCACGGTCTGTTCG	56.6	NM_007808.4
	R: TGCCCTTTCTCCCTTCTTC		
*COX2*	F: TGACAGTCCACCTACTTACAAT	55.2	AF378830.1
	R: CTCCACCAATGACCTGATAT		
*β-actin*	F: ACTGCCGCATCCTCTTCCTC	56.7	AY550069
	R: CTCCTGCTTGCTGATCCACATC		
*TFAM*	F: AGCAGGTTTACGAGGAAGCA	59.31	NM_204100.1
	R: TTGAAGCCACTTCGAGGTCT		
*FABP4*	F: ATGTGCGACCAGTTTGTG	50.8	NM_024406
	R: TTTGCCATCCCACTTCTG		
*FATP1*	F: CGGTGCTGTTACGAGTGA	61.5	NM_001039602
	R: CACGGCGTTGGAATACTT		
*FAT*	F: CTGGGAAGGTTACTGCGATTT	52.9	DQ323177
	R: TTCACGGTCTTACTGGTCTGG		
*UCP2*	F: GTGGATGCCTACAGGACCAT	60.1	AF287144.1
	R: GAAGTGACAGGGGACGTTGT		
*ACC*	F: CCATATGGACCTCTTCTTGT	58.8	AB004329.1
	R: AAGTCCTTTCGCCTTGCAGT		

F^a^: Forward primer.

R^b^: Reverse primer.
